# On the Performance of Energy Harvesting Non-Orthogonal Multiple Access Relaying System with Imperfect Channel State Information over Rayleigh Fading Channels

**DOI:** 10.3390/s19153327

**Published:** 2019-07-29

**Authors:** Tran Manh Hoang, Nguyen Le Van, Ba Cao Nguyen, Le The Dung

**Affiliations:** 1 Faculty of Radio Electronics, Le Quy Don Technical University, Hanoi 11917, Vietnam; 2 Faculty of Telecommunications Services, Telecommunications University, Khanh Hoa 650000, Vietnam; 3 Division of Computational Physics, Institute for Computational Science, Ton Duc Thang University, Ho Chi Minh City 758307, Vietnam; 4 Faculty of Electrical and Electronics Engineering, Ton Duc Thang University, Ho Chi Minh City 758307, Vietnam

**Keywords:** NOMA, energy harvesting, amplify-and-forward, imperfect CSI, successive interference cancellation (SIC)

## Abstract

In this paper, we propose a non-orthogonal multiple access (NOMA) relaying system, where a source node communicates simultaneously with multiple users via the assistance of the best amplify-and-forward (AF) relay. The best relay is selected among *N* relays which are capable of harvesting the energy from radio frequency (RF) signals. We analyze the performance of the proposed NOMA relaying system in the conditions of imperfect channel state information (CSI) and Rayleigh fading by deriving the exact expressions of the outage probability (OP) and the approximate expression of the ergodic capacities of each user and the whole system. We also determine the optimal energy harvesting duration which minimizes the OP. Numerical results show that, for the same parameter settings, the performance of the proposed NOMA relaying system, especially the ergodic capacity of the whole system, outperforms that of the orthogonal-multiple-access (OMA) relaying system. Monte-Carlo simulations are used to validate the correctness of the analytical results.

## 1. Introduction

Nowadays, the Internet of Things (IoT) has received increasing attention from both industry and academia. It is considered an important mean for wireless connections in the fourth industrial revolution. IoT is also being used in the fourth generation (4G) mobile communications and will be applied to the fifth generation (5G). In order to support a large multiuser system such as IoT, the non-orthogonal multiple access (NOMA) is a very potential technique due to its high bandwidth efficiency [1,2]. Moreover, compared with conventional orthogonal multiple access (OMA) systems, such as time division multiple access (TDMA), code division multiple access (CDMA), orthogonal frequency division multiple access (OFDMA), the NOMA systems offer better fairness among users, even for users with weak channel conditions such as the cell-edge users. The fundamentals of the NOMA system can be found in Reference [3] while a study of NOMA system in cellular communication with machine-to-machine in IoT is given in Reference [4].

Recently, the power supply for terminal devices in wireless networks has become an important matter and has attracted much interest from researchers. Besides using the optimal power allocation for the fifth generation (5G) and sixth generation (6G) networks (6G network will start to enter the market by 2026 [5]) to reduce the power consumption [6], another promising method to improve the lifetime of communication devices is to generate electric power from some external energy sources such as solar, wind, and radio frequency (RF) signal to charge the batteries. Unfortunately, natural energy sources are not suitable for small-size mobile devices and in some cases they cannot be used in the healthcare monitoring networks and the sensor networks with real-time requirements. In contrast, the RF energy is often available due to its increased power density and availability, and is independent on environmental conditions, including weather, climate, and temperature. As the result, the RF energy harvesting (EH), also called simultaneous wireless information and power transfer (SWIPT), has been widely used compared with other kinds of energy harvesting techniques [7,8,9]. SWIPT has been applied not only in the point-to-point systems but also in relaying systems because deploying relays can improve the amount of harvested energy and the coverage area of wireless networks. The authors of References [10,11] investigated information and energy receiver architecture for SWIPT networks. Reference [11] especially considered a non-linear energy harvesting model which described the practical system well.

To prolong network lifetime and improve the spectral utilizing efficiency, NOMA is combined with SWIPT [12]. In Reference [13], the authors investigated the tradeoff among the energy efficiency, fairness, harvested energy, and system sum rate of NOMA systems in power domain. Investigation of an integrated wireless communication system including NOMA, full-duplex relaying, and energy harvesting techniques was conducted in Reference [14]. The authors of References [15,16] studied the system performance of cooperative NOMA systems and derived the expressions of outage probability in the conditions of perfect successive interference cancellation (SIC) and perfect channel state information (CSI). In Reference [17], the near users which are close to the base station will harvest the RF energy and forward signals to far users. The analysis results showed that if the time switching ratio in NOMA system with SWIPT is appropriately chosen, the diversity gain will not be impaired. The authors of Reference [18] proposed a NOMA system where source node communicates with two users via the assistance of the best relay with the RF energy harvesting capability. The exact expressions of the outage probability and throughput were used as the criteria to evaluate the system performance. The effects of power allocation and time switching ratio on the performance of multi-user NOMA system were investigated in Reference [19]. Specifically, the authors derived the outage probability expression and determined the optimal power allocation coefficient for two NOMA power allocation policies, namely NOMA with fixed power allocation (F-NOMA) and cognitive radio inspired NOMA (CR-NOMA). It was shown that when a reasonable power allocation coefficient is selected, higher system performance can be achieved in comparison with the conventional multi-user system.

We observe that all previous works only mentioned the case of perfect CSI and used only one relay to forward signals to multiple users. Moreover, although the partial relay selection has been widely studied in conventional wireless systems, it has not been analyzed in NOMA systems. Another observation is that the NOMA systems perform superimposing signals in power domain, thus they always require CSI to allocate power for all users. However, due to variation in the communication quality of wireless environment, the imperfect CSI may happen [20,21]. Perfect CSI exists if and only if the amount of feedback CSI from users to the base station is large and the length of the pilot sequences which are used to estimate channel is very long. Unfortunately, these conditions rarely happen in practice. Therefore, investigation of the impact of imperfect CSI on the relay selection and power allocation is vitally important to the design of practical NOMA systems.

Motivated by the above issues, in this paper we propose a downlink NOMA relaying system with partial relaying selection. In this system, source node transmits superposition modulated signals to multiple users via the assistance of the best relay. The best relay is chosen from a set of relays which are capable of harvesting RF energy and grouped by their locations. Based on the feedback CSI from all users, the source node performs power allocation and chooses the best communication link. The main contributions of this paper can be summarized as follows:

- We overcome the limitation of current multiple access techniques and the energy demand of wireless networks by proposing the downlink NOMA relaying system where the best relay is selected from a set of multiple RF energy harvesting relays.
- We study the system performance in terms of the outage probability and the ergodic capacity of each user and the whole system in the condition of imperfect CSI and Rayleigh fading. The imperfection of the CSI is modeled by the correlation coefficient and its impact on the system performance is investigated by using both analysis and simulation approaches. We also compare the outage performance and the ergodic capacity of the proposed NOMA relaying system with those of OMA relaying system.
- We determine the optimal time switching ratio to balance between the energy harvesting and the signal processing so that the outage probability can be minimized. All analysis results are validated by simulation results.

The rest of this paper is organized as follows. Section 2 describes the proposed downlink NOMA relaying system with partial relaying selection and time switching (TS) protocol. The analysis of the outage probability and ergodic capacity of the proposed system are presented in Section 3 and Section 4, respectively. Section 5 shows numerical results to evaluate the system performance. Finally, the conclusions are given in Section 6.

For the sake of clarity, the frequently used mathematical notations together with their descriptions are summarized in Table 1.

## 2. System Model

**Figure 1:**
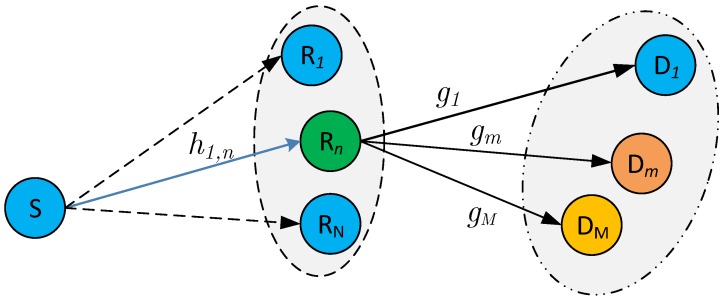
System model of downlink non-orthogonal multiple access (NOMA) relaying system with simultaneous wireless information and power transfer (SWIPT).

Figure 1 illustrates the proposed downlink NOMA relaying system. In this system, source node S transmits the signals which are coded and superposed in power domain to multiple users $D_{m}$, m∈{1,⋯,M}, via the assistance of the best relay which is selected from a set of relays $R_{n}$, n∈{1,⋯,N}. The direct link S-$D_{m}$ is assumed not available because the distance between S and $D_{m}$ is larger than the coverage area of S or due to deep shadow fading.

We consider that all nodes are equipped with single antenna and operate in half-duplex mode. All channels between S and $R_{n}$ and between $R_{n}$ and $D_{m}$ are influenced by block fading, that is, the symbol rate is larger than channel varying rate so that it can be considered as constant over each symbol duration. The communication links from S to $R_{n}$ and from $R_{n}$ to $D_{m}$ are respectively modeled as complex Gaussian distributions with zero mean and variances $\Omega_{1,n}$ and $\Omega_{R_{n}D_{m}}$, that is, $h_{1,n}∼\mathrm{CN}\left(0,\Omega_{1,n}\right)$ and $g_{m}∼\mathrm{CN}\left(0,\Omega_{R_{n}D_{m}}\right)$. The Additive White Gaussian Noise (AWGN) at the relays and users are $w_{R_{n}}∼\mathrm{CN}\left(0,\sigma_{R_{n}}^{2}\right)$ and $w_{D_{m}}∼\mathrm{CN}\left(0,\sigma_{D_{m}}^{2}\right)$, respectively. Because of the time varying characteristic of wireless channel, its coherent time may be altered when the feedback delay is larger than the transmission block period of a symbol. Thus, the received CSI is always outdated at the transmitter, which often happens in practice [23,24].

We denote $\rho_{i}$, $0\leq \rho_{i}\leq 1$, $i\in \left\{1,2\right\}$, as the correlation coefficients between the past channel $h_{1,n}$ and the current channel ${\hat{h}}_{1,n}$, similarly for ${\hat{g}}_{m}$ and $g_{m}$. These coefficients can be considered as the measurements of the fluctuation rate of wireless channels and are related solely to the time delay. Based on the Markov chain, the relationship between ${\hat{h}}_{1,n}$ and $h_{1,n}$ and between ${\hat{g}}_{m}$ and $g_{m}$ can be presented as [25]

(1) $$\begin{array}{c} {\hat{h}}_{1,n}=\rho_{1}h_{1,n}+\sqrt{1-\rho_{1}^{2}}\varepsilon_{1,n}, \end{array}$$

(2) $$\begin{array}{c} {\hat{g}}_{m}=\rho_{2}g_{m}+\sqrt{1-\rho_{2}^{2}}\varepsilon_{m}, \end{array}$$

where $\varepsilon_{1,n}$ and $\varepsilon_{m}$ are the circular symmetric complex Gaussian random variables which can be modeled as $\varepsilon_{1,n}∼CN\left(0,\;\sigma^{2}\right)$ and $\varepsilon_{m}∼CN\left(0,\;\sigma^{2}\right)$, respectively.

In this paper, a partial relay selection (PRS) scheme [26] is used to select the best relay from a set of relays. According to the PRS scheme, S continuously monitors the gain of S–$R_{n}$ channels by using the feedback signal and selects the communication link that gives the largest instantaneous channel gain, that is,

(3) $$\begin{array}{c} \gamma_{b}=\arg\;\;\max_{n=1,\;2\cdots ,N}\gamma_{1,n}, \end{array}$$

where $\gamma_{1,n}$ is the instantaneous SINR of S-$R_{n}$ link.

The advantage of using PRS scheme is that the system configuration is simpler and easier than using the full relay selection (FRS) scheme [27]. In addition, the results in Reference [28] show that both PRS and FRS schemes have the same average channel capacity in a high SNR regime and the outage probability of PRS is higher than that of FRS when the number of relays is more than 2. On the other hand, FRS scheme may not applicable for multiple-user systems because the distances from the best relay to users are different, thus the calculation complexity of FRS scheme is significantly increased with the number of users.

After a link from the source node to the best relay is established, the transmission period *T* for communication process is spitted into two parts (in this system, we use the time-division multiple access (TDMA) scheme). According to the time switching (TS) protocol [29], a time duration αT is used for EH. The remaining time duration (1−α)T is divided into two equal time sub-slots, which are used for the information transmission. The first half (1−α)T/2 is used for the information transmission from source node to the relay and the remaining half (1−α)T/2 is used for the information transmission from the relay to the user. It should be noted that the case α=1 is not considered in this paper because when the energy harvesting time duration takes the whole transmission period *T*, i.e., the relay does not process any signals, the basic role in signal forwarding of the relay is eliminated [30]. Hence, we only consider the time switching ratio α in the range 0≤α<1. Then, the harvested energy of the selected relay in time duration αT is expressed as [29]

(4) $$\begin{array}{c} E_{h}=\alpha T\eta P_{S}\max_{n=1,\cdot \cdot \cdot ,N}{\left|{\hat{h}}_{1,n}\right|}^{2}, \end{array}$$

where η is the energy conversion efficiency coefficient which varies from 0 to 1 and closely depends on the quality of energy harvesting electric circuitry, $P_{S}$ is the transmission power of S.

In our proposed relaying system, since the harvest-use (HU) architecture is used, the relay does not need an energy buffer to store the harvested energy. Since all amounts of harvested energy during EH phase is consumed by $R_{n}$ for signal transmission from R to $D_{m}$, from (4), the transmission power of the best relay is given by

(5) $$\begin{array}{c} P_{R}=\frac{E_{h}}{(1-\alpha )T/2}=\frac{2\alpha \eta P_{S}}{1-\alpha }\max_{n=1,\cdot \cdot \cdot ,N}{\left|{\hat{h}}_{1,n}\right|}^{2}. \end{array}$$

According to the NOMA technique in power domain, during the first time sub-slot $\frac{1-\alpha }{2}$, source node transmits the superimposed signal $x_{S}=\sum_{m=1}^{M}\sqrt{P_{S}a_{m}}x_{m}$, where $x_{m}$ and $a_{m}$ are the signal and power allocation coefficient of *m*th user, respectively. At the end of this time sub-slot, the received signal at $R_{n}$ is

(6) $$\begin{array}{c} y_{R}^{n}={\hat{h}}_{1,b}\sum_{m=1}^{M}\sqrt{a_{m}P_{S}}x_{m}+w_{R_{n}}, \end{array}$$

where ${\hat{h}}_{1,b}=\max_{n=1,\cdot \cdot \cdot ,N}{\left|{\hat{h}}_{1,n}\right|}^{2}$.

In the remaining second time slot $\frac{1-\alpha }{2}$, the relay employs the AF protocol to broadcast $y_{R}^{n}$ to all users after multiplying it with an amplifying factor *G*. To keep the output power constraint at relay, it is required that $E\{∥Gy_{R}^{n}{∥}^{2}\}=P_{R}$, where $P_{R}$ is given in (5), thus the amplifying factor *G* is given by

(7) $$\begin{array}{c} G=\sqrt{\frac{2\alpha \eta P_{S}{\left|{\hat{h}}_{1,b}\right|}^{2}}{\left(1-\alpha \right)(P_{S}|{\hat{h}}_{1,b}{|}^{2}+\sigma_{R}^{2})}}\approx \sqrt{\frac{2\alpha \eta }{\left(1-\alpha \right)}}. \end{array}$$

Therefore, the received signal at $D_{m}$ in the case of perfect SIC is expressed as

(8) $$\begin{array}{cc} y_{D_{m}}=G{\hat{h}}_{1,b}{\hat{g}}_{m}\sqrt{a_{m}P_{S}}x_{m} & +\underset{\mathrm{signals}\;\mathrm{of}\;\mathrm{other}\;\mathrm{users}}{\underbrace{G{\hat{h}}_{1,b}{\hat{g}}_{m}\sum_{j=m+1}^{M}\sqrt{a_{j}P_{S}}x_{j}}}+\underset{\mathrm{noise}}{\underbrace{G{\hat{g}}_{m}w_{R_{n}}+w_{D_{m}}}}, \end{array}$$

where ${\hat{g}}_{m}$ denotes the channel coefficient between $R_{n}$ and $D_{m}$.

The received signals at the best relay and each user is comprised of the desired signal and the signals of other users, which are treated as the interferences. Hence, to mitigate the negative effect of the inter-user interference, successive interference cancellation (SIC) method is applied.

For the downlink communication considered in this paper, the optimal SIC algorithm performs decoding signals in an order of increasing channel gain [31] ($|g_{D_{1}}{|}^{2}\leq |g_{D_{2}}{|}^{2}\leq \cdots \leq |g_{D_{m}}{|}^{2}\leq {\left|g_{D_{M}}\right|}^{2}$). To ensure the fairness among all users, the power allocation coefficients are assumed to be $a_{1}\geq a_{2}\geq \cdots a_{m}\geq a_{M}$, with $\sum_{m=1}^{M}a_{m}=1$. Hence, at the $D_{j}$, the signal of $D_{m}$, j<m, will be detected and then be removed from the received signal by SIC method. Specifically, $D_{j}$ first decodes symbol $x_{m}$ while treating $x_{j}$ as noise.

Then, the SINR of symbol $x_{m}$ at $D_{j}$ is given by

(9) $$\begin{array}{c} \gamma_{m,j}^{D}=\frac{G^{2}a_{m}P_{S}|{\hat{h}}_{1,b}{|}^{2}{\left|{\hat{g}}_{j}\right|}^{2}}{G^{2}\sum_{j=m+1}^{M}a_{j}P_{S}|{\hat{h}}_{1,b}{|}^{2}|{\hat{g}}_{j}{|}^{2}+G^{2}{\left|{\hat{g}}_{j}\right|}^{2}\sigma_{R}^{2}+\sigma_{D_{m}}^{2}}, \end{array}$$

where $j\in \left\{1,\ldots ,m\right\}$ and m≠M.

At $D_{j}$, SIC will be performed until all signals of $D_{m}$ are decoded successfully. Thus, the required SINR at $D_{m}$ to successfully decode the signal by itself is given by

(10) $$\begin{array}{c} \gamma_{m}^{D}=\frac{G^{2}a_{m}P_{S}|{\hat{h}}_{1,b}{|}^{2}{\left|{\hat{g}}_{m}\right|}^{2}}{G^{2}\sum_{j=m+1}^{M}a_{j}P_{S}|{\hat{h}}_{1,b}{|}^{2}|{\hat{g}}_{m}{|}^{2}+G^{2}{\left|{\hat{g}}_{m}\right|}^{2}\sigma_{R}^{2}+\sigma_{D_{m}}^{2}}. \end{array}$$

We should note that the last user $D_{M}$ needs to decode all signals of other users before decoding its signals. Consequently, the SINR for $D_{M}$ to decode its own signals can be expressed as

(11) $$\begin{array}{c} \gamma_{M}^{D}=\frac{G^{2}a_{M}P_{S}|{\hat{h}}_{1,b}{|}^{2}{\left|g_{M}\right|}^{2}}{G^{2}{\left|g_{M}\right|}^{2}\sigma_{R}+\sigma_{D_{M}}^{2}}. \end{array}$$

## 3. Outage Probability Analysis

In this section, we derive the exact closed-form expression of the outage probability, taking into consideration the imperfect CSI and partial relay selection. It is well-known that the event that $D_{j}$ can decode the signals of $D_{m}$ successfully is

(12) $$\begin{array}{c} \Delta_{m,j}=\left\{\frac{G^{2}a_{m}P_{S}|{\hat{h}}_{1,b}{|}^{2}{\left|{\hat{g}}_{j}\right|}^{2}}{G^{2}\sum_{j=m+1}^{M}a_{j}P_{S}|{\hat{h}}_{1,b}{|}^{2}|{\hat{g}}_{j}{|}^{2}+G^{2}{\left|{\hat{g}}_{j}\right|}^{2}\sigma_{R}^{2}+\sigma_{D_{m}}^{2}}>\gamma_{\mathrm{th}j}\right\}, \end{array}$$

where $\gamma_{\mathrm{th}j}=2^{\frac{2r}{1-\alpha }}-1$ is the predefined outage threshold. This threshold is served as the protected value of the SINR to ensure the quality of service of the system and satisfy the target data rate *r* of $D_{j}$.

Let us denote $X=|{\hat{h}}_{1,b}{|}^{2}$ and $Z=|{\hat{g}}_{j}{|}^{2}$. Without loss of generality, we assume that the temperature noise $\sigma_{R}^{2}=\sigma_{D_{m}}^{2}=\sigma^{2}$. Thus from (12), we can rewrite $\Delta_{m,j}$ as

(13) $$\begin{array}{c} \Delta_{m,j}=\left\{\frac{G^{2}a_{m}P_{S}XZ}{G^{2}\sum_{j=m+1}^{M}a_{j}P_{S}XZ+G^{2}Z+\sigma^{2}}>\gamma_{\mathrm{th}j}\right\}. \end{array}$$

From (13) and after some manipulations, we can rewrite (13) as

(14) $$\begin{array}{c} \Delta_{m,j}\overset{\left(\lambda \right)}{\;=}\left\{X>\theta_{j},\;\;\;Z>\frac{\theta_{j}}{G^{2}\left(X-\theta_{j}\right)}\right\}, \end{array}$$

where $\theta_{j}=\frac{\gamma_{\mathrm{th}j}}{P_{S}\left(a_{m}-\sum_{j=m+1}^{M}a_{j}\gamma_{\mathrm{th}j}\right)}$, step λ holds when the condition $a_{m}>\sum_{j=1+m}^{M}a_{j}\gamma_{\mathrm{th}j}$ is satisfied. It should be noticed that $\theta_{j}=\frac{\gamma_{\mathrm{th}j}}{P_{S}\left(a_{m}-\sum_{j=m+1}^{M}a_{j}\gamma_{\mathrm{th}j}\right)}$ is a constant and depends on the power allocation coefficient and the target data rate of $D_{j}$.

The outage event occurs at $D_{j}$ when it fails to decode its own signal or unsuccessfully performs SIC for the signals of $D_{m}$ [32], i.e., $\Lambda_{m,j}=\gamma_{m,j}^{D}<\gamma_{\mathrm{th}j},1\leq j\leq m$. Outage probability of the system occurs when the maximum SNRs at $D_{j}$ falls below the threshold to decode signal. Thus, we have

(15) $$\begin{array}{c} P_{out}^{j}=\mathrm{Pr}\left(\gamma_{m,j}^{D}\leq \gamma_{\mathrm{th}j}\right)=1-\mathrm{Pr}\left(\gamma_{m,j}^{D}>\gamma_{\mathrm{th}j}\right),\;\;\;\;1\leq j\leq m. \end{array}$$

(16) $$\begin{array}{c} P_{out}^{j}=1-\mathrm{Pr}\left\{\Delta_{m,1}\cap \Delta_{m,2}\cap \ldots \cap \Delta_{m,m}\;\right\}, \end{array}$$

where $\Delta_{m,j}$ is the complementary in the set of $\Lambda_{m,j}$.

The condition in (14) always occurs, i.e., the outage probability is equal to one, if $a_{m}\leq \sum_{j=1+m}^{M}a_{j}\gamma_{\mathrm{th}j}$. Hence, we need to allocate more power for $D_{m}$ to satisfy the following condition

(17) $$\begin{array}{c} a_{m}>\sum_{j=1+m}^{M}a_{j}\gamma_{\mathrm{th}j}. \end{array}$$

Let us denote $\theta^{*}=\max\left(\theta_{1},\theta_{2},\cdots ,\theta_{m}\right)$ [33], then the outage probability $P_{out}^{j}$ of $D_{j}$ can be reformulated as

(18) $$\begin{array}{c} P_{out}^{j}=1-\mathrm{Pr}\left\{Z>\frac{\theta^{*}}{G^{2}\left(X-\theta^{*}\right)},\;\;X>\theta^{*}\right\}. \end{array}$$

Using the conditional probability property [34] with respect to *X*, and applying the law of joint CDF, we have

(19) $$\begin{array}{c} P_{out}^{j}=1-\int_{\theta^{*}}^{\infty }\left[1-F_{Z}\left(\frac{\theta^{*}}{G^{2}\left(x-\theta^{*}\right)}\right)\right]f_{X}\left(x\right)dx. \end{array}$$

To calculate the expression of the outage probability in (19), we first derive the CDF of *Z* and the PDF of *X* as follows.

When the *n*th relay is selected as the best relay, the PDF of order statistic with respect to $|h_{1,b}{|}^{2}$ in a set of *N* relays is obtained by using the binomial Newton expansion [35], that is,

(20) f|h1,b|2(x)=NF|h1,i|2(x)N−1f|h1,i|2(x)=∑n=1NNn(−1)n−1nΩ1,nexp−nxΩ1,n,

where Nn=n!n!(N−n)!, *N* and *n* are non-negative integers, $f_{|h_{1,i}{|}^{2}}\left(x\right)=\frac{1}{\Omega_{1,i}}\exp\left(-\frac{x}{\Omega_{1,i}}\right)$ and $F_{|h_{1,i}{|}^{2}}\left(x\right)=1-\exp\left(-\frac{x}{\Omega_{1,i}}\right)$ are respectively the CDF and PDF of $|h_{1,i}{|}^{2}$, which is the channel gain of each link from source node to relay. According to the probability theory, the PDFs of $|{\hat{h}}_{1,b}{|}^{2}$ and $|h_{1,b}{|}^{2}$ which are respectively denoted by $f_{|{\hat{h}}_{1,b}{|}^{2}}\left(\hat{x}\right)$ and $f_{|h_{1,b}{|}^{2}}\left(x\right)$ can be calculated by using the joint PDF, i.e., $f_{|{\hat{h}}_{1,b}{|}^{2}}\left(\hat{x}\right)=\int_{0}^{\infty }f_{|{\hat{h}}_{1,b}{|}^{2},{\left|h_{1,b}\right|}^{2}}\left(\hat{x},x\right)dx$. Another way to calculate the joint PDF of $|{\hat{h}}_{1,b}{|}^{2}$ is based on the properties of conditional probability, that is,

(21) $$\begin{array}{c} f_{|{\hat{h}}_{1,b}{|}^{2}}\left(\hat{x}\right)=\int_{0}^{\infty }f_{|{\hat{h}}_{1,b}{|}^{2}\left|\right|h_{1,b}{|}^{2}}\left(\hat{x}|x\right)f_{|h_{1,b}{|}^{2}}\left(x\right)dx, \end{array}$$

where

(22) $$\begin{array}{c} f_{|{\hat{h}}_{1,b}{|}^{2}\left|\right|h_{1,b}{|}^{2}}\left(\hat{x}|x\right)=\frac{f_{|{\hat{h}}_{1,i}{|}^{2},{\left|h_{1,i}\right|}^{2}}\left(\hat{x},x\right)}{f_{|h_{1,i}{|}^{2}}\left(x\right)}. \end{array}$$

Using the joint PDF which is given in ([36], Equation (9.389)), we can rewrite the numerator of (22) as

(23) $$\begin{array}{c} f_{|{\hat{h}}_{1,i}{|}^{2},{\left|h_{1,i}\right|}^{2}}\left(\hat{x},x\right)=\frac{\exp\left(-\frac{\left(\hat{x}+x\right)}{\left(1-\rho^{2}\right)\Omega_{1,n}}\right)}{\left(1-\rho^{2}\right)\Omega_{1,n}^{2}}I_{0}\left(\frac{2\rho \sqrt{\hat{x}x}}{\left(1-\rho^{2}\right)\Omega_{1,n}}\right), \end{array}$$

where $I_{0}\left(x\right)$ is the modified zero order Bessel function of the first kind [22].

Without loss of generality, all correlation coefficients are assumed to have the same values, that is, $\rho =\rho_{1}=\rho_{2}$. Substituting (20), (22), and (23) into (21), after using the equation $\int_{0}^{\infty }e^{-\alpha z}I_{0}\left(2\sqrt{\beta z}\right)dz=\left(1/\alpha \right)\exp\left(\beta /\alpha \right)$ which is given in ([22], Equation (6.614.3)), and then perform some manipulations, we have the PDF of *X* in the case of imperfect CSI as

(24) f|h^1,b|2(x^)=∑n=1NNnn(−1)n−1Ω1,nΨ(ρ,n)exp−nx^Ω1,nΨ(ρ,n),

where $\Psi \left(\rho ,n\right)=1+\left(n-1\right)\left(1-\rho^{2}\right)$.

From (24), the CDF of $|{\hat{h}}_{1,b}{|}^{2}$ is given by

(25) F|h^1,b|2(x^)=1−∑n=1NNn(−1)n−1exp−nx^Ω1,nΨ(ρ,n).

Based on the result of order statistics which is provided in ([34], Equation (7.14), p. 246), and after some similar calculations as above, the PDF of the ordered variable *Z* is expressed as

(26) f|^^gj|2(z)=∑j=1MMj(−1)j−1jΩzΨ(ρ,j)exp−jzΩzΨ(ρ,j),

where $\Psi \left(\rho ,j\right)=1+\left(j-1\right)\left(1-\rho^{2}\right)$.

From (26), we can derive the CDF of $|{\hat{g}}_{i}{|}^{2}$ as

(27) F|g^i|2(z^)=1−∑j=1MMj(−1)j−1exp−jz^ΩzΨ(ρ,j).

Plugging (27) and (24) into (19), and after some manipulations, we obtain the expression of the outage probability as

(28) Poutj=1−∑j=1MMj(−1)j−1∑n=1NNnn(−1)n−1Ω1,nΨ(ρ,n)∫θ*∞exp−jθ*ΩzΨ(ρ,j)G2(x−θ*)−nx^Ω1,nΨ(ρ,n)dx.

Let $u=x-\theta^{*}$, (28) becomes

(29) Poutj=1−∑j=1MMj(−1)j−1∑n=1NNnn(−1)n−1Ω1,nΨ(ρ,n)exp−nθ*Ω1,nΨ(ρ,n)×∫0∞exp−jθ*ΩzΨ(ρ,j)G2u−nuΩ1,nΨ(ρ,n)du.

Using ([22], Equation (3.324)), we can rewrite the exact closed-form expression of the outage probability as in (30), where $K_{1}\left(.\right)$ denotes the modified first order Bessel function of the second kind.

(30) Poutj=1−∑j=1MMj(−1)j−1∑n=1NNn(−1)n−1Ω1,nexp−nθ*Ω1,nΨ(ρ,n)×4njθ*ΩzΨ(ρ,j)Ω1,nΨ(ρ,n)G2K14njθ*ΩzΨ(ρ,j)Ω1,nΨ(ρ,n)G2.

From the expression of the outage probability which is given in (30), we can see that when the outdated CSI happens, the outage performance is a function of ρ.

## 4. Ergodic Capacity Analysis

In this section, we analyze the ergodic capacity of the proposed NOMA relaying system in comparison with that of the OMA relaying system. Due to the fact that the hardware complexity and performance degradation of the NOMA system is directly proportional to the number of users, we also set the number of users be equal to three for both NOMA and OMA systems as used in [37]. For the OMA system, we consider orthogonal frequency division multiple access (OFDMA). According to the Shannon theory, the instantaneous rate of $D_{m}$ is given by

(31) $$\begin{array}{c} R_{\mathrm{NOMA}}^{m\mathrm{th}}=\frac{1-\alpha }{2}\log_{2}\left(1+\gamma_{m}^{D}\right). \end{array}$$

From (10), when the transmission power is high, we can approximate the required SINR at $D_{m}$ as

(32) $$\begin{array}{c} \gamma_{m}^{D}\approx \frac{G^{2}a_{m}P_{S}|{\hat{h}}_{1,b}{|}^{2}{\left|{\hat{g}}_{m}\right|}^{2}}{G^{2}\sum_{j=m+1}^{M}a_{j}P_{S}|{\hat{h}}_{1,b}{|}^{2}{\left|{\hat{g}}_{m}\right|}^{2}+\sigma_{D_{m}}^{2}}. \end{array}$$

Substituting (32) into (31), we have

(33) $$\begin{array}{cc} R_{\mathrm{NOMA}}^{m\mathrm{th}} & \approx \frac{1-\alpha }{2}\log_{2}\left(1+\frac{G^{2}a_{m}P_{S}|{\hat{h}}_{1,b}{|}^{2}{\left|{\hat{g}}_{m}\right|}^{2}}{G^{2}\sum_{j=m+1}^{M}a_{j}P_{S}|{\hat{h}}_{1,b}{|}^{2}{\left|{\hat{g}}_{m}\right|}^{2}+\sigma_{D_{m}}^{2}}\right)\\ & =\frac{1-\alpha }{2}\log_{2}\left(\frac{G^{2}P|{\hat{h}}_{1,b}{|}^{2}{\left|{\hat{g}}_{m}\right|}^{2}+1}{G^{2}\sum_{j=m+1}^{M}a_{j}P|{\hat{h}}_{1,b}{|}^{2}{\left|{\hat{g}}_{m}\right|}^{2}+1}\right), \end{array}$$

where $P=\frac{P_{S}}{\sigma_{D_{m}}^{2}}$.

Based on the properties of the logarithmic function, we can rewrite (33) as

(34) $$\begin{array}{cc} R_{\mathrm{NOMA}}^{m\mathrm{th}}= & \underset{I_{1}}{\underbrace{\frac{1-\alpha }{2}E\left\{\log_{2}\left(1+G^{2}P|{\hat{h}}_{1,b}{|}^{2}{\left|{\hat{g}}_{m}\right|}^{2}\right)\right\}}}\\ & -\underset{I_{2}}{\underbrace{\frac{1-\alpha }{2}E\left\{\log_{2}\left(1+G^{2}\sum_{m=1}^{M-1}a_{m}P|{\hat{h}}_{1,b}{|}^{2}{\left|{\hat{g}}_{m}\right|}^{2}\right)\right\}}}, \end{array}$$

then solve its components by using the partial integration, i.e.,

(35) $$\begin{array}{cc} I_{u}= & {\left\{\log_{2}\left(1+\Gamma_{u}\right)\left[F_{\Gamma_{u}}\left(x_{u}\right)-1\right]\right\}}_{0}^{\infty }-\frac{1}{2\ln2}\int_{0}^{\infty }\frac{1}{1+x_{u}}\left[F_{\Gamma_{u}}\left(x_{u}\right)-1\right]dx_{u}\\ = & \frac{1}{2\ln2}\int_{0}^{\infty }\frac{1}{1+x_{u}}\left[1-F_{\Gamma_{u}}\left(x_{u}\right)\right]dx_{u}, \end{array}$$

where $F_{\Gamma_{u}}\left(x_{u}\right)$ is the CDF of random variable $\Gamma_{u}$ with u∈{1,2}, $\Gamma_{1}=G^{2}P|{\hat{h}}_{1,b}{|}^{2}{\left|{\hat{g}}_{m}\right|}^{2}$, and $\Gamma_{2}=G^{2}\sum_{m=1}^{M-1}a_{m}P|{\hat{h}}_{1,b}{|}^{2}{\left|{\hat{g}}_{m}\right|}^{2}$.

Using the condition probability, we have CDF of $\Gamma_{1}$ as

(36) $$\begin{array}{cc} F_{\Gamma_{1}}\left(x_{1}\right) & =\mathrm{Pr}(G^{2}P|{\hat{h}}_{1,b}{|}^{2}|{\hat{g}}_{m}{|}^{2}\leq x_{1})\\ & =\int_{0}^{\infty }\mathrm{Pr}\left(|{\hat{g}}_{m}{|}^{2}\leq \frac{x_{1}}{G^{2}P{\left|{\hat{h}}_{1,b}\right|}^{2}}\right)f_{|{\hat{h}}_{1,b}{|}^{2}}d{\left|{\hat{h}}_{1,b}\right|}^{2}. \end{array}$$

From (24) and (27) we can calculate $F_{\Gamma_{1}}\left(x_{1}\right)$ as

(37) FΓ1(x1)=1−∑j=1MMj(−1)j−1∑n=1NNn(−1)n−1Ω1,n×4njx1ΩzΨ(ρ,j)Ω1,nΨ(ρ,n)PG2K14njx1ΩzΨ(ρ,j)Ω1,nΨ(ρ,n)PG2.

Similarly, for $F_{\Gamma_{2}}\left(x_{2}\right)$, we have

(38) FΓ2(x2)=1−∑j=1MMj(−1)j−1∑n=1NNn(−1)n−1Ω1,n×4njx2ΩzΨ(ρ,j)Ω1,nΨ(ρ,n)bPG2K14njx2ΩzΨ(ρ,j)Ω1,nΨ(ρ,n)bPG2.

where $b=\sum_{m=1}^{M-1}a_{m}$.

Replacing (38) into (35), we obtain $I_{1}$ as

(39) I1=12ln2∑j=1MMj(−1)j−1∑n=1NNn(−1)n−1Ω1,n×∫0∞11+x1A(n,j)x1K1A(n,j)x1dx1,

where $A=\frac{4}{\Omega_{z}\Psi \left(\rho ,j\right)\Omega_{1,n}\Psi \left(\rho ,n\right)PG^{2}}$.

Based on ([22], Equation (9.343)), we can rewrite (39) as

(40) $$\begin{array}{c} I_{1}=\frac{1-\alpha }{2\sqrt{2}\ln2}\int_{0}^{\infty }\frac{1}{1+x_{1}}G_{0\;\;2}^{2\;\;0}\left(\left.\frac{x_{1}}{\Omega_{z}\Psi \left(\rho ,j\right)\Omega_{1,n}\Psi \left(\rho ,n\right)PG^{2}}\right|\begin{array}{c} \frac{3}{4},-\;\frac{1}{4} \end{array}\right)dx_{1}. \end{array}$$

Then, using ([22], Equation (7.811.5)) and after some manipulations, we have

(41) $$\begin{array}{c} I_{1}=\frac{1-\alpha }{2\sqrt{2}\ln2}G_{1\;\;3}^{3\;\;1}\left({\left.\frac{1}{\Omega_{z}\Psi \left(\rho ,j\right)\Omega_{1,n}\Psi \left(\rho ,n\right)PG^{2}}\right|}_{0,\;\;\frac{3}{4},\;-\;\frac{1}{4}}^{0}\right), \end{array}$$

where $G_{pq}^{mn}\left(x{|}_{b_{s}}^{a_{r}}\right)$ is the Meijer’s G-Function ([22], Equation (9.3)).

Plugging (38) into (35), and doing similar manipulations which were used to derive $I_{1}$, we obtain

(42) $$\begin{array}{c} I_{2}=\frac{1-\alpha }{2\sqrt{2}\ln2}G_{1\;\;3}^{3\;\;1}\left({\left.\frac{1}{\Omega_{z}\Psi \left(\rho ,j\right)\Omega_{1,n}\Psi \left(\rho ,n\right)bPG^{2}}\right|}_{0,\;\;\frac{3}{4},\;-\;\frac{1}{4}}^{0}\right), \end{array}$$

To compare the ergodic capacities of the NOMA and OMA systems, we let β be the bandwidth which is assigned for $D_{1}$ and (1−β)/2 be the remaining bandwidth which is assigned for $D_{2}$ and $D_{3}$, where (0<β<1) and the whole bandwidth is 1Hz. From ([38], Equation (7.4)), we can extend the achievable end-to-end ergodic capacity of the OFDMA system with three users as

(43) $$\begin{array}{cc} R_{\mathrm{OMA}}= & \frac{1-\alpha }{2}\beta \log_{2}\left(1+\gamma_{{\mathrm{SRD}}_{1}}\right)\\ & +\frac{\left(1-\alpha )(1-\beta \right)}{4}\log_{2}\left(1+\gamma_{{\mathrm{SRD}}_{2}}\right)\\ & +\frac{\left(1-\alpha )(1-\beta \right)}{4}\log_{2}\left(1+\gamma_{{\mathrm{SRD}}_{3}}\right), \end{array}$$

where $\gamma_{{\mathrm{SRD}}_{m}},m\in \left\{1,2,3\right\}$ denotes the instantaneous SINR of each user, which is computed as

(44) $$\begin{array}{cc} \gamma_{{\mathrm{SRD}}_{1}} & =\frac{G^{2}P_{S}^{\mathrm{OMA}}|{\hat{h}}_{1,b}{|}^{2}{\left|{\hat{g}}_{1}\right|}^{2}}{\beta (G^{2}|{\hat{g}}_{1}{|}^{2}\sigma_{R}^{2}+\sigma_{D_{1}}^{2})}, \end{array}$$

(45) $$\begin{array}{cc} \gamma_{{\mathrm{SRD}}_{2}} & =\frac{2G^{2}P_{S}^{\mathrm{OMA}}|{\hat{h}}_{1,b}{|}^{2}{\left|{\hat{g}}_{2}\right|}^{2}}{\left(1-\beta \right)(G^{2}|{\hat{g}}_{2}{|}^{2}\sigma_{R}^{2}+\sigma_{D_{2}}^{2})}, \end{array}$$

(46) $$\begin{array}{cc} \gamma_{{\mathrm{SRD}}_{3}} & =\frac{2G^{2}P_{S}^{\mathrm{OMA}}|{\hat{h}}_{1,b}{|}^{2}{\left|{\hat{g}}_{3}\right|}^{2}}{\left(1-\beta \right)(G^{2}|{\hat{g}}_{3}{|}^{2}\sigma_{R}^{2}+\sigma_{D_{3}}^{2})}, \end{array}$$

where $P_{S}^{\mathrm{OMA}}=P_{S}/3$ is the equal power allocated for the signal transmission from S to each user $D_{m}$ ([38], p. 146). The factor $\frac{1-\alpha }{2}$ appears in (31) and (43) because source node transmits its signals to all users in two time slots of the transmission period *T*.

## 5. Numerical Results

In this section, we provide the numerical results to evaluate the system performance in terms of the outage probability (OP) and ergodic capacity of the proposed EH-NOMA relaying system with three users. We also determine the optimal time switching ratio to minimize the OP and compare the ergodic capacities of the proposed EH-NOMA relaying system with EH-OMA relaying system. Regarding to the evaluating method, we use the common approach in this field, that is, to drive a closed-form mathematical expression to model the system performance and then compare the analysis results with Monte-Carlo simulation results to validate the derived mathematical expressions. Unlike previous works, which only considered EH-NOMA systems with two users and under perfect CSI, our paper focuses on the theoretical analysis of an EH-NOMA system with more than two users, taking into account the effects of AF relaying protocol and the feedback delay of wireless channels on the system performance. Since, there are not many similar parameters, it may be an unfair comparison between our proposed EH-NOMA relaying system with previous NOMA relaying systems. Therefore, we use the same system model of the proposed EH-NOMA relaying sytem but replace the NOMA with OMA to demonstrate the benefits of utilizing the NOMA technique in the proposed EH relaying system. Unless otherwise stated, the parameter settings of EH-NOMA and EH-OMA relaying systems are summarized in Table 2. It is noticed that the average SNR is defined as the ratio of the transmission power of source S to the variance of AWGN, that is, $\mathrm{SNR}=P_{S}/\sigma^{2}$, ranging from 0 dB to 40 dB.

**Figure 2:**
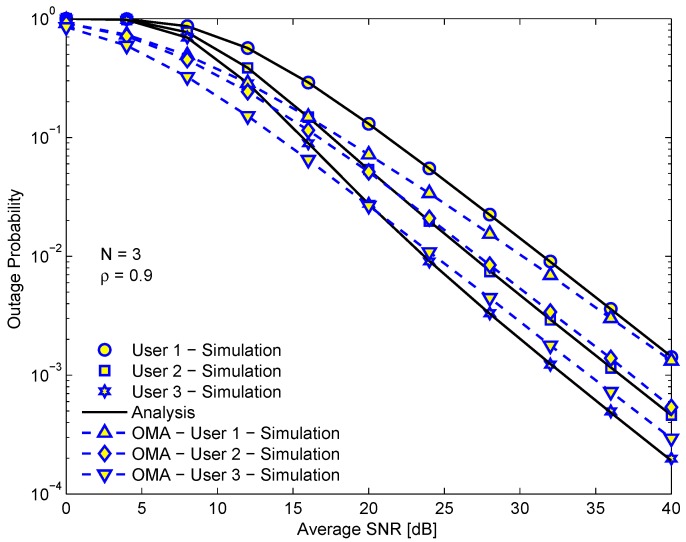
The outage probability of each user in energy harvesting (EH)-NOMA and EH-OMA relaying systems versus the average SINR. ρ=0.9, the number of relays *N* = 3.

Figure 2 shows the outage probability of each user versus the average SINR in dB. The outage probability of the EH-NOMA relaying system is also compared with that of EH-OMA relaying system. Firstly, we can see that the OP of $D_{3}$ is lowest among all users while the OP of $D_{1}$ is highest. The reason is that the channel gain from R to $D_{3}$ is highest (the decay of the magnitude power signal is proportional to the squared distance in multipath fading) because $D_{3}$ is the closest user to R while $D_{1}$ is the farthest one. Another important observation is that the OPs of $D_{2}$ and $D_{3}$ in the EH-NOMA relaying system are better than those of $D_{2}$ and $D_{3}$ in the EH-OMA relaying system, while the OP of $D_{1}$ in the EH-OMA relaying system is better than in the NOMA relaying system. However, the gap is insignificant because the number of time slots for the transmission in the EH-OMA relaying system is higher than in the EH-NOMA relaying system, thus the probability that outage evens happen in the EH-OMA relaying system is also higher than in the EH-NOMA relaying system. On the other hand, the outage threshold of the OMA user is $\gamma_{\mathrm{th}}^{\mathrm{OMA}}=2^{\frac{2r}{v(1-\alpha )}}-1$, where v∈{β,(1−β)/2}. In contrast, the outage threshold of the NOMA user is $\gamma_{\mathrm{th}}=2^{\frac{2r}{\left(1-\alpha \right)}}-1$. Then, obviously the outage threshold of the OMA user is obviously higher than that of the NOMA user. However, the OP not only depends on the outage threshold but also on the received SINR at user. In addition, we also see that in the low SINR regime (less than 15 dB), the OPs of all OMA users always outperform those of NOMA users. However, in the high SINR regime (larger 15 dB) only the OP of $D_{1}$ in the EH-OMA relaying system is better than that in the EH-NOMA relaying system. We can also see in Figure 2 that the diversity gain of all users is equal to one.

**Figure 3:**
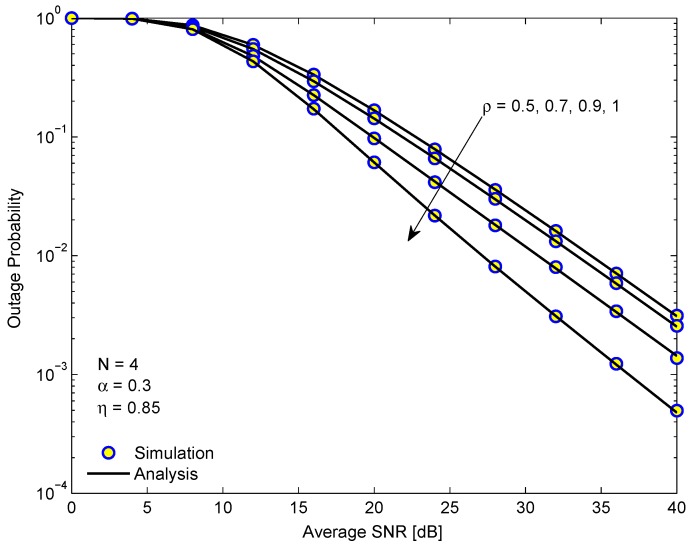
The outage probability of $D_{1}$ in the EH-NOMA relaying system versus the average SINR for different correlation coefficients.

Figure 3 plots the OP of $D_{1}$ in the EH-NOMA relaying system versus the average SINR in dB for different channel correlation coefficients ρ. Firstly, we see that higher ρ reduces the OP, but the reduction is not remarkable for small ρ. The improvement in OP is only significant when ρ is near to 1. We should remind that ρ indicates the correlation degree between the transmission channel and the feedback channel in time coherent at the transmitter. The analysis results are in excellent agreement with the simulation ones, confirming the correctness of our mathematical analysis.

**Figure 4:**
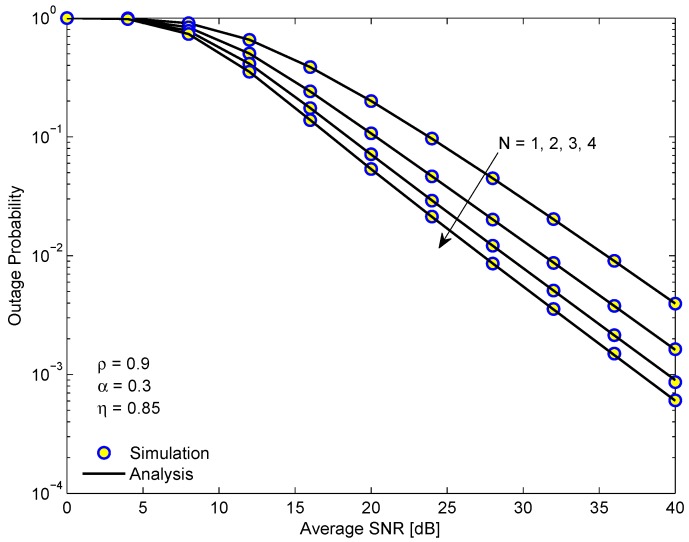
The outage probability of $D_{1}$ in the EH-NOMA relaying system versus the average SINR for different numbers of relays.

Figure 4 illustrates the OP of $D_{1}$ in the NOMA system versus the average SINR in dB for different numbers of relays *N*. From Figure 4, we see that when the number of relays increases, the outage performance of the system is improved. It is because increasing the number of relays will provide more opportunity for selecting the connection links from source node to relay, which not only makes the achievable decoding performance better but also increases the amount of harvested energy. In addition, the diversity gains is not significantly improved with *N* because the diversity order of PRS scheme is always equal to one.

**Figure 5:**
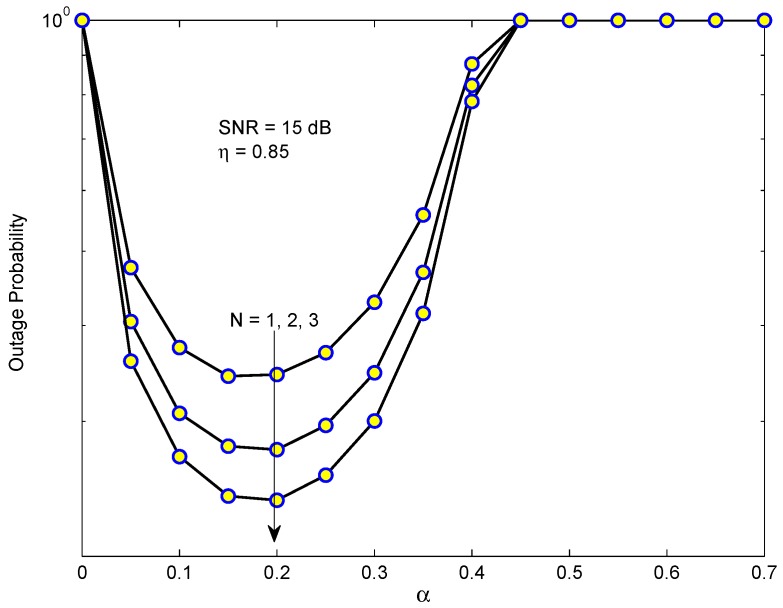
The outage probability of $D_{1}$ in EH-NOMA relaying versus the time switching ratio α for different number of relays.

Figure 5 presents the OP of $D_{1}$ in the EH-NOMA relaying system versus time switching ratio α for different numbers of relays. The values of α range from 0 to 0.7 while SINR remains at 15 dB. Firstly, we see that there exists an optimal value of α which minimizes the OP. Moreover, the minimum value of OP depends on the number of relays *N*, i.e., as *N* is higher the minimal OP becomes smaller. The reason is that when *N* increases, the SINR of the first hop will be better because the PRS method is used. Another important observation is that the optimal value of α which minimizes the OP is approximately 0.2 regardless of the number of relays.

**Figure 6:**
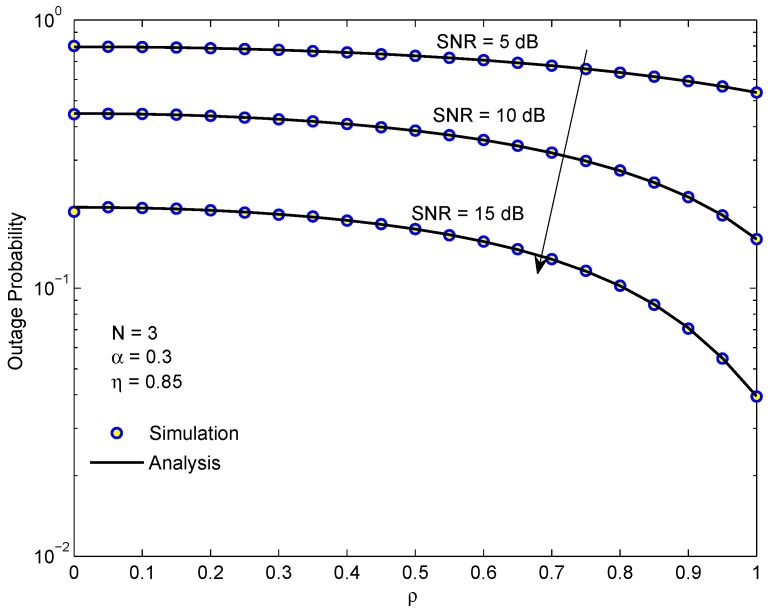
The outage probability of $D_{1}$ in the EH-NOMA relaying system versus the correlation coefficient for different average SINRs.

Figure 6 demonstrates the OP of $D_{1}$ in the EH-NOMA relaying system versus the correlation coefficient ρ for different average SINR. We can see that the OP reduces as ρ increases. In the worst case ρ=0, the instantaneous CSI at the transmission time does not correlate with the instantaneous CSI at the relay-selection time or at the power-allocation time. In contrast, in the best case ρ=1, the instantaneous CSI at the transmission time closely correlates with the instantaneous CSI at the relay-selection time or at the power-allocation time. The improvement in the CSI leads to better power allocation and signal processing of the system. Figure 6 also shows that when ρ<0.8, the enhancement of OP is not significant and the system performance is only improved when the correlation coefficient ρ is close to 1.

**Figure 7:**
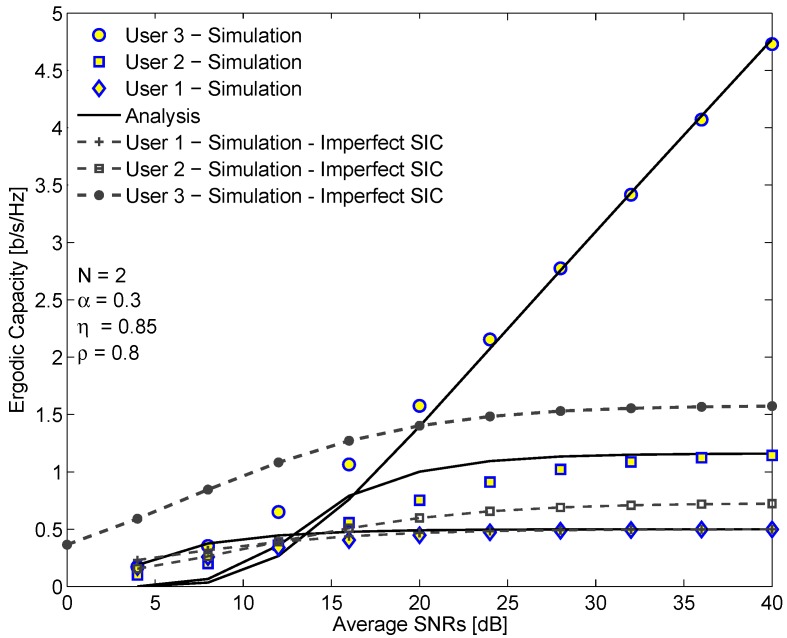
The ergodic capacity of each user and the ergodic capacity in EH-NOMA relaying system versus the average SINR.

Figure 7 depicts the ergodic capacity of each user in EH-NOMA relaying system versus the average SINR in dB. As observed from Figure 7, the ergodic capacity of $D_{3}$ outperforms the ergodic capacities of $D_{1}$ and $D_{2}$. Moreover, the ergodic capacities of $D_{1}$ and $D_{2}$ increase slightly in the low SINR region and is saturated in the high SINR region. In contrast, the ergodic capacity of $D_{3}$ increases exponentially with respect to the SINR. This reason is that $D_{1}$ does not use SIC but only detects the signal of itself. Meanwhile, $D_{2}$ must use the first-order SIC first and then $D_{3}$ uses the second-order SIC. Thus, the impact of interference on $D_{1}$ is higher than $D_{2}$ and $D_{3}$. However, there exists the trade-off between the complexity and the achievable ergodic capacity of the system. We also see a good match between the analysis results and the simulation results, especially in the high SINR regime. On the other hand, the ergodic capacity in the case of perfect SIC is compared with that in the case of imperfect SIC. We can see that the ergodic capacity in case imperfect SIC is lower. Moreover, the gap between them increases with the SINR. It is because when SINR increases, the interference caused by imperfect SIC also increases. Therefore, the SINR as well as the ergodic capacity become slowly higher. Another feature is that the ergodic capacity of $D_{1}$ remains the same in both cases because $D_{1}$ does not use SIC when decoding the signals.

**Figure 8:**
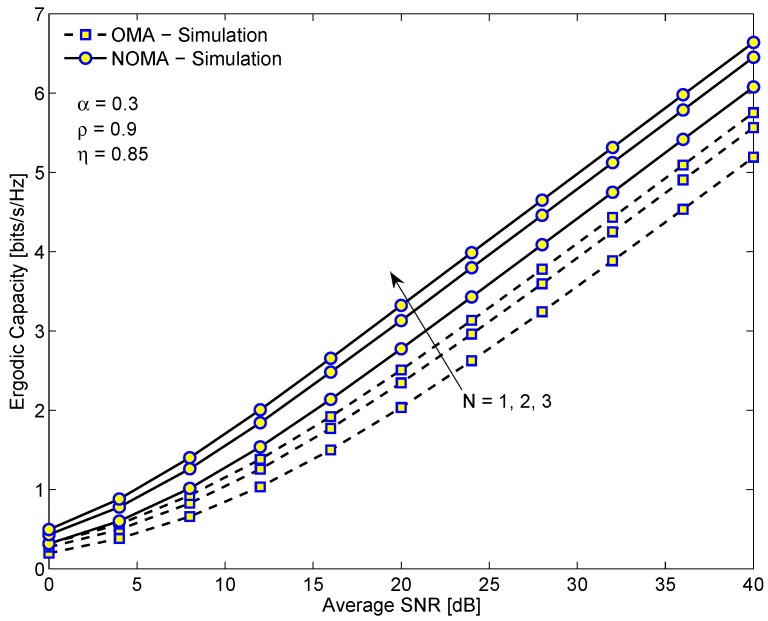
The comparison of the ergodic capacities of the EH-NOMA relaying system and the EH-OMA relaying system versus the average SINR for different numbers of relays.

Figure 8 provides the simulation results of the ergodic rate of NOMA and OMA systems versus the average SINR in dB. From Figure 8, we see that the ergodic rate of NONA system is always higher than the OMA system as the number of relays increases. It is because the NOMA system uses the whole bandwidth for each user while the OMA system uses individual bandwidth for each user, resulting in higher spectrum usage efficiency. Another important observation is that when *N* gets higher, the difference gap of the ergodic capacities of these two systems does not increase linearly. Thus, we do not need to use a large number of relays for partial relay selection scheme because it may increase the complexity of the system but not significantly enhance its performance.

## 6. Conclusions

In this paper, we propose a downlink NOMA relaying system with the best RF energy harvesting relay and investigate the impact of CSI imperfection on the performance of the proposed NOMA relaying system over Rayleigh fading channel. Specifically, we provide detailed derivations of the exact closed-form expression of OP and the approximate expression of the ergodic capacity of the proposed NOMA relaying system. Based on the expression of the OP, the optimal energy harvesting duration which minimizes the OP in the condition of imperfect CSI can be determined. The results show that imperfect CSI significantly reduces the system performance. In addition, we show that the spectrum efficiency of our proposed NOMA relaying system outperforms that of the OMA relaying system in the same parameter settings. All analysis results are in excellent agreement with the simulation results, confirming the correctness of the mathematical analysis. The proposed EH-NOMA relaying system can support the communication for multiple users through the best relay without relying on the external power supply. Thus it can be applied in surveillance sensor networks for disaster detection or in Internet of Things (IoT) where installing fixed power lines or frequent battery replacement for a large number of nodes may be not convenient. Using the results in this paper, we can choose an appropriate time switching ratio to balance between the energy harvesting and signal processing so that the outage probability of the proposed EH-NOMA relaying system system can be reduced upto 76.32%. Moreover, compared with the EH-OMA relaying system, the OP of the proposed EH-NOMA relaying system is 9.41% lower and the ergodic capacity is 17.64% higher at the average SNR = 40 dB.

## Figures and Tables

**Table 1:** The mathematical notations used in this paper.

Notation	Description
$F_{U}\left(u\right)$	Cumulative distribution function (CDF)
$f_{U}\left(u\right)$	Probability density function (PDF)
$\mathrm{CN}(\mu ,\sigma^{2})$	Circularly symmetric complex Gaussian distribution *X* with mean μ and variance $\sigma^{2}$
$\gamma_{\mathrm{th}}$	Predefined outage threshold
$E\left\{\cdot \right\}$	Expectation operator
$K_{n}\left(\cdot \right)$	Second order Bessel function *n* [22]
$I_{0}\left(\cdot \right)$	Modified zero order Bessel function of first kind [22]
α	Time switching ratio
η	Energy conversion efficiency
ρ	Channel correlation coefficient
*T*	Transmission period

**Table 2:** Parameter settings of EH-NOMA and EH-OMA relaying systems.

Description	EH-NOMA	EH-OMA
Allocated transmission power	$P_{1}=0.7P_{S},P_{2}=0.2P_{S}$, $P_{3}=0.1P_{S}$	$P_{i}=P_{S}/3$
Bandwidth	β for $D_{1}$, (1−β)/2 for $D_{2}$ and $D_{3}$	*B* = 1 Hz for all users
Target data rate	r=0.5 bpcu	
Time switching ratio	α=0.3	
Average channel gain	$\Omega_{1,n}=1$, $\Omega_{R_{n}D_{1}}=2$, $\Omega_{R_{n}D_{2}}=3$, $\Omega_{R_{n}D_{3}}=6$	
Energy conversion efficiency	η=0.85	

## References

[1] Liu Y., Qin Z., Elkashlan M., Ding Z., Nallanathan A., Hanzo L. (2017). Nonorthogonal multiple access for 5G and beyond. IEEE Commun. Surv. Tutor..

[2] Liu Y., Qin Z., Elkashlan M., Nallanathan A., McCann J.A. (2017). Non-orthogonal multiple access in large-scale heterogeneous networks. IEEE J. Sel. Areas Commun..

[3] Wang B., Dai L., Xiao M. (2018). Millimeter Wave NOMA. Encyclopedia of Wireless Networks.

[4] Lv T., Ma Y., Zeng J., Mathiopoulos P.T. (2018). Millimeter-Wave NOMA Transmission in Cellular M2M Communications for Internet of Things. IEEE Internet Things J..

[5] David K., Berndt H. (2018). 6G Vision and Requirements: Is There Any Need for Beyond 5G?. IEEE Veh. Technol. Mag..

[6] Long D.N. (2018). Resource Allocation for Energy Efficiency in 5G Wireless Networks. EAi Endorsed Trans. Ind. Netw. Intell. Syst..

[7] Le T.D., Tran M.H., Nguyen T.T., Choi S.G. Analysis of partial relay selection in NOMA systems with RF energy harvesting. Proceedings of the International Conference on Recent Advances in Signal Processing, Telecommunications & Computing (SigTelCom).

[8] Tran M.H., Vu V.S., Nguyen C.D., Pham T.H. (2018). Optimizing Duration of Energy Harvesting for Downlink NOMA Full-Duplex over Nakagami-*m* fading channel. AEu-Int. J. Electron. Commun..

[9] Tran H.V., Kaddoum G. (2018). RF wireless power transfer: Regreening future networks. IEEE Potential.

[10] Tran H.V., Kaddoum G. (2019). Robust Design of AC Computing-Enabled Receiver Architecture for SWIPT Networks. IEEE Wirel. Commun. Lett..

[11] Tran H.V., Kaddoum G., Truong T.K. (2018). Resource allocation in SWIPT networks under a nonlinear energy harvesting model: Power efficiency, user fairness, and channel nonreciprocity. IEEE Trans. Veh. Technol..

[12] Bariah L., Muhaidat S., Al-Dweik A. (2019). Error Probability Analysis of NOMA-based Relay Networks with SWIPT. IEEE Commun. Lett..

[13] Moltafet M., Azmi P., Mokari N., Javan M.R., Mokdad A. (2018). Optimal and fair energy efficient resource allocation for energy harvesting-enabled-PD-NOMA-based HetNets. IEEE Trans. Wirel. Commun..

[14] Guo C., Zhao L., Feng C., Ding Z., Chen H. (2019). Energy Harvesting Enabled NOMA Systems with Full-duplex Relaying. IEEE Trans. Veh. Technol..

[15] Sun R., Wang Y., Wang X., Zhang Y. (2016). Transceiver Design for Cooperative Non-Orthogonal Multiple Access Systems with Wireless Energy Transfer. IET Commun..

[16] Han W., Ge J., Men J. (2016). Performance Analysis for NOMA Energy Harvesting Relaying Networks with Transmit Antenna Selection and Maximal-Ratio Combining over Nakagami-*m* Fading. IET Commun..

[17] Liu Y., Ding Z., Elkashlan M., Poor H.V. (2016). Cooperative non-orthogonal multiple access with simultaneous wireless information and power transfer. IEEE J. Sel. Areas Commun..

[18] Tran M.H., Nguyen T.T., Nguyen H.H., Pham T.H. (2018). Performance analysis of decode-and-forward partial relay selection in NOMA systems with RF energy harvesting. Wirel. Netw..

[19] Yang Z., Ding Z., Fan P., Al-Dhahir N. (2017). The Impact of Power Allocation on Cooperative Non-orthogonal Multiple Access Networks with SWIPT. IEEE Trans. Wirel. Commun..

[20] Dutta B., Budhiraja R., Koilpillai D.R. (2017). Limited-feedback low-encoding complexity precoder design for downlink of FDD multi-user massive MIMO systems. IEEE Trans. Commun..

[21] Hoydis J., Brink S.T., Debbah M. (2017). Massive MIMO in the UL/DL of cellular networks: How many antennas do we need?. IEEE J. Sel. Areas Commun..

[22] Zwillinger D. (2014). Table of Integrals, Series, and Products.

[23] Firag A., Smith P.J., Suraweera H.A., Nallanathan A. (2011). Performance of beamforming in correlated MISO systems with estimation error and feedback delay. IEEE Trans. Commun..

[24] Tran M.H., Tran X.N., Nguyen T., Le T.D. (2018). Performance Analysis of MIMO SWIPT Relay Network with Imperfect CSI. Mob. Netw. Appl..

[25] Suraweera H.A., Smith P.J., Shafi M. (2010). Capacity limits and performance analysis of cognitive radio with imperfect channel knowledge. IEEE Trans. Veh. Commun..

[26] Bletsas A., Khisti A., Reed D.P., Lippman A. (2006). A simple cooperative diversity method based on network path selection. IEEE J. Sel. Areas Commun..

[27] Tran T.D., Kong H.Y. (2013). Performance analysis of incremental amplify-and-forward relaying protocols with nth best partial relay selection under interference constraint. Wirel. Pers. Commun..

[28] Tran T.D., Duong Q.T., Costa D.B., Vo N.Q.B., Elkashlan M. (2015). Proactive relay selection with joint impact of hardware impairment and co-channel interference. IEEE Trans. Commun..

[29] Nasir A.A., Zhou X., Durrani S., Kennedy R.A. (2013). Relaying protocols for wireless energy harvesting and information processing. IEEE Trans. Wirel. Commun..

[30] Ju M.C., Kang K.M., Hwang K.S., Jeong C. (2015). Maximum Transmission Rate of PSR/TSR Protocols in Wireless Energy Harvesting DF-Based Relay Networks. IEEE J. Sel. Areas Commun..

[31] Pedersen K.I., Kolding T.E., Seskar I., Holtzman J.M. Practical implementation of successive interference cancellation in DS/CDMA systems. Proceedings of the ICUPC—5th International Conference on Universal Personal Communications.

[32] Men J., Ge J., Zhang C. (2016). Performance analysis of non-orthogonal multiple access for relaying networks over Nakagami-*m* fading channels. IEEE Trans. Veh. Technol..

[33] Men J., Ge J., Zhang C. (2017). Performance analysis for downlink relaying aided non-orthogonal multiple access networks with imperfect CSI over Nakagami-*m* fading. IEEE Access.

[34] Papoulis A., Pillai S.U. (2002). Probability, Random Variables, and Stochastic Processes.

[35] Xiong J., Tang Y., Ma D., Xiao P., Wong K.K. (2015). Secrecy performance analysis for TAS-MRC system with imperfect feedback. IEEE Trans. Inf. Forensics Secur..

[36] Simon M.K., Alouini M.S. (2000). Digital Communication Over Generalized Fading Channels: A Unified Approach to Performance Analysis.

[37] Bariah L., Muhaidat S., Al-Dweik A. (2018). Error Probability Analysis of Non-Orthogonal Multiple Access over Nakagami-*m* Fading Channels. IEEE Trans. Commun..

[38] Benjebbour A., Saito K., Li A., Kishiyama Y., Nakamura T. (2016). Non-Orthogonal Multiple Access (NOMA): Concept and Design. Signal Processing for 5G: Algorithms and Implementations.

